# Qingjie Fuzheng Granule Inhibited the Migration and Invasion of Colorectal Cancer Cells by Regulating the lncRNA ANRIL/let-7a/TGF-*β*1/Smad Axis

**DOI:** 10.1155/2020/5264651

**Published:** 2020-06-29

**Authors:** Ling Zhang, Jianxin Liu, Shan Lin, Jingzhuang Tan, Bin Huang, Jiumao Lin

**Affiliations:** ^1^Academy of Integrative Medicine, Fujian University of Traditional Chinese Medicine, Fuzhou, Fujian 350122, China; ^2^Fujian Key Laboratory of Integrative Medicine on Geriatrics, Fujian University of Traditional Chinese Medicine, Fuzhou, Fujian 350122, China

## Abstract

Qingjie Fuzheng granule (QFG) promotes cancer cell apoptosis and ameliorates intestinal mucosal damage caused by 5-fluorouracil. However, the antitumor role of QFG in colorectal cancer (CRC) progression remains unclear. In this study, the growth of HCT-8 and HCT116 cells incubated with various concentrations of QFG for 24 and 48 h was evaluated using MTT assays; their abilities of migration and invasion were investigated through wound healing and Transwell assays. The expression of lncRNA ANRIL, let-7a, and the TGF-*β*1/Smad signaling pathway components was assessed using real-time PCR and western blotting. The results elicited that QFG significantly suppressed the growth of HCT-8 and HCT116 cells; the half-maximal inhibitory concentrations (IC_50_) of QFG for HCT-8 and HCT116 cells for 48 h were 1.849 and 1.608 mg/mL, respectively. The abilities of wound healing, migration, and invasion of HCT-8 and HCT116 cells were dose-dependently decreased by QFG treatment for 24 h, respectively. QFG decreased the expression of lncRNA ANRIL, TGF-*β*1, phosphorylated (p)-Smad2/3, Smad4, and N-cadherin and upregulated the expression of let-7a in HCT-8 and HCT116 cells. Collectively, our data demonstrated that QFG inhibited the metastasis of CRC cells by regulating the lncRNA ANRIL/let-7a/TGF-*β*1/Smad axis, indicating that they might serve as an adjunctive medicine for CRC treatment.

## 1. Introduction

Globally, colorectal cancer (CRC) is considered the second leading cause of cancer-related deaths [1]. Clinical treatment options for metastatic colon cancer are limited, and this is the most common cause of high mortality rates for CRC [2]. During early metastatic progression, cancer cells undergo morphological changes called epithelial-mesenchymal transition (EMT). EMT leads to epithelial cells losing their adhesion and polarity properties and gaining a mesenchymal phenotype [3]. Subsequently, the cancer cells disseminate from the primary sites to distant organs [3]. Emerging evidence suggests that a family of long noncoding RNAs (lncRNAs) comprising >200 nucleotides with little to unknown protein-coding potential plays a vital role in cancer metastasis [4].

Ever-increasing evidence suggests that abnormal expression or dysfunction of lncRNAs is involved in the malignant behaviors of human cancers. Among them, the lncRNA antisense noncoding RNA in the INK4 locus (ANRIL), also identified as CDKN2B antisense RNA1, promotes cell migration, invasion, lymphangiogenesis, and lymphatic metastasis, all of which are related to the survival of CRC patients [5, 6]. Furthermore, the lncRNA ANRIL is a molecular sponge for let-7a and negatively regulates the expression of let-7a. It also promotes chemoresistance, represses tumorigenicity, and improves cell proliferation and migration via downregulation of let-7a expression in various cancers [7–9]. The transforming growth factor-*β*1 (TGF-*β*1)/Smad pathway is closely involved in cancer metastasis and is crucial downstream signaling pathway of the lncRNA ANRIL and let-7a. In this pathway, TGF-*β*1 phosphorylates TGF-*β*1 receptor type I (TGF-*β*R I), which subsequently phosphorylates the intracellular proteins Smad2/3 followed by Smad2/3 binding to Smad4; this leads to increasing N-cadherin expression and decreasing E-cadherin expression, thus promoting the progression of EMT [10, 11].

Traditional Chinese medicine (TCM) is clinically effective for treating cancer [12]. Qingjie Fuzheng granule (QFG) is a TCM formulation consisting of *Oldenlandia diffusa* (Willd.) Roxb., *Astragalus membranaceus* (Fisch.) Bge. var. *mongholicus* (Bge.) Hsiao, *Hordeum vulgare* L., and *Scutellariae Barbatae* D. Don. Studies showed that QFG played vital roles in the promotion of cell apoptosis in hepatocellular carcinoma by regulating the death receptor pathway and mitochondrion-dependent pathway [13] and the inhibition of cell growth in CRC cells through the regulation of the PI3K/AKT and ERK signaling pathways [14]. Moreover, QFG was reported to ameliorate 5-fluorouracil- (5-FU-) induced intestinal mucositis and diarrhea via inhibiting inflammatory responses and attenuating the damage of jejunum tissue [15]. Clinically, it acts as an adjunctive medicine in the mFOLFOX4 regimen for advanced CRC patients [16]. However, the role(s) and potential molecular mechanism(s) of the antitumor effects of QFG in CRC progression remain largely unknown. The present study evaluated the antimetastasis effects of QFG and aimed to elucidate the underlying mechanism(s) of the antitumor effects of QFG using wound healing and Transwell assays, real-time PCR, and western blotting.

## 2. Materials and Methods

### 2.1. Cell Culture

HCT-8 and HCT116 cells (KeyGen Biotech Co., Ltd., Nanjing, China) were maintained in RPMI-1640 medium (Gibco, Thermo Fisher Scientific Inc., Waltham, MA, USA) supplemented with 10% fetal bovine serum (FBS, Gibco, Thermo Fisher Scientific Inc.), 100 U/mL penicillin, and 100 *μ*g/mL streptomycin (Hyclone Laboratories Inc., South Logan, UT, USA) and incubated in a humidified incubator under a 5% CO_2_ atmosphere at 37°C.

### 2.2. Preparation of QFG

QFG was obtained from the College of Pharmacy of Fujian University of Traditional Chinese Medicine (Fuzhou, Fujian, China). We combined 1.25 kg *Oldenlandia diffusa* (Willd.) Roxb., 1.25 kg *Astragalus membranaceus* (*Fisch*) *Bge*. *var*. *mongholicus* (*Bge*) *Hsiao*, 1.25 kg *Hordeum vulgare* L., and 1.25 kg *Scutellariae Barbatae* D. Don with 50 L of 70% ethanol, soaking for 30 min. Then, the Chinese herbal compounds were extracted with a refluxing method and filtered twice. Subsequently, filtered liquid was evaporated on a rotary evaporator and the resulting solution was dried into a powder which was further prepared into granules by spraying. They were all dissolved in PBS (Hyclone, Logan, UT, USA) to a final concentration of 200 mg/mL, sonicated for 30 min, passed through 0.45 *μ*m filters, and stored at −20°C.

### 2.3. MTT Assay

HCT-8 and HCT116 cells were incubated into 96-well plates with a density of 1 × 10^5^ cells/mL. Following attachment, the cells of HCT-8 and HCT116 were treated with 0, 0.25, 0.5, 1, 1.5, or 2 mg/mL QFG for 24 h or 48 h. Thereafter, 100 *μ*L of 3-(4,5-dimethylthiazol-2-yl)-2,5-diphenyltetrazolium bromide (MTT) (0.5 mg/mL) (Solarbio Science & Technology Co., Ltd, Beijing, China) was added to each well and the plate was incubated for 4 h at 37°C. The supernatants were decanted and 100 *μ*L of DMSO was added to each well to dissolve the MTT formazan precipitate. Absorbance at 570 nm was detected via an ELX800 microplate reader (BioTek Instruments, Inc., Winooski, VT, USA).

### 2.4. Wound Healing Assay

HCT-8 and HCT116 cells were seeded into six-well plates. When the confluence reached 90%, a white tip was used to draw scratches; the floating cells were removed using PBS. The attached cells were treated with 0, 0.5, 1, 2 mg/mL QFG for 24 h. The area of wound was visually assessed at 0, 12, and 24 h via a phase contrast microscope (Leica Microsystems GmbH, Germany) at a magnification of 100×. A decrease in scratch width indicated migration.

### 2.5. Transwell Assay

Cell migration was further assessed via Transwell cell culture chambers with 8 *μ*m pore filters (Corning Life Sciences, Corning, NY, USA). Following an incubation with QFG (0, 0.5, 1, and 2 mg/mL) for 24 h, surviving HCT-8 and HCT116 cells were further seeded into the upper chambers at a density of 5 × 10^4^ cells/chamber. Culture medium containing 10% FBS was added to the lower chambers as a chemoattractant. Some cells migrated towards the complete medium within 12 h and were stained with crystal violet for 15 min at room temperature. Cells in the upper chamber that had not migrated were removed using wet cotton swabs. To calculate the average number of migrated (stained) cells per field, three fields were randomly selected using a phase contrast microscope (Leica Microsystems GmbH, Germany) at a magnification of 200× to count the stained cells. The setup for cell invasion assays was the same as that for cell migration, except that the upper chambers contained Matrigel matrix (BD Biosciences, Franklin Lakes NJ, USA).

### 2.6. RNA Extraction and Reverse Transcription-Quantitative PCR (RT-qPCR) Analysis

Total RNA was extracted from HCT-8 and HCT116 cells incubated with QFG (0, 0.5, 1, or 2 mg/mL) for 24 h using Trizol (Thermo Fisher Scientific Inc.). The cDNA was reverse transcribed using Oligo (dT) or special let-7a RT-primers with HiScript II 1st Strand cDNA Synthesis Kit (Vazyme Biotech Co., Ltd, Nanjing, China). The obtained cDNA was used as the template to detect the expression of lncRNA ANRIL and let-7a using SYBR™ Select Master Mix kit (Thermo Fisher Scientific, Inc., Waltham, MA, USA). Polymerase chain reactions were performed using an ABI 7500 Fast PCR system with the following reaction sequence: initial denaturation 95°C for 5 min, 40 cycles of 95°C for 10 sec, and 60°C for 30 sec. The respective internal controls for lncRNA ANRIL and let-7a were GAPDH and U6. The relative expression of lncRNA ANRIL and let-7a was analyzed using the 2^−ΔΔCt^ method [17].

### 2.7. Western Blot Analysis

Total protein was extracted from HCT-8 and HCT116 cells after a 24 h incubation with QFG (0, 0.5, 1, and 2 mg/mL), using cell lysis buffer (Pierce; Thermo Fisher Scientific, Inc., Waltham, MA, USA) containing protease and phosphatase inhibitors, then placed on ice for 30 min. Equal amounts of protein were separated using 10% SDS-PAGE gels and transferred to PVDF membranes (Millipore Corporation, Billerica, MA, USA). Nonspecific protein binding was blocked by incubating the membranes with 5% nonfat dry milk for at least 1 h at room temperature. Then, the proteins were probed overnight at 4°C using primary antibodies against TGF-*β*1 (Cat no: 3711), phosphorylated (p-) Smad2/3 (Cat no: 8828), Smad2/3 (Cat no: 8685), and Smad4 (Cat no: 38454) (all from Cell Signaling Technology, Inc., Beverly, MA, USA; all diluted 1 : 1,000); against N-cadherin (Cat no: ab18203; diluted 1 : 1,000) and E-cadherin (Cat no: ab1416; diluted 1 : 2,000) (both from Abcam, Cambridge, UK); and against *β*-actin (Cat no: 66009-1-Ig; Proteintech Group Inc., Chicago, IL, USA; diluted 1 : 5,000). After three washes with TBS/Tween-20, the membranes were incubated with the appropriate HRP-conjugated secondary antibodies (Cat no: SA00001-1 and SA00001-2; Proteintech Group Inc.; diluted 1 : 10,000) at room temperature for 1 h. After three washes with TBS/Tween-20, immunoreactive bands were visualized using Image lab 3.0 software (Bio-Rad Laboratories Inc., Hercules, CA, USA) and enhanced chemiluminescence (Yuheng Biotech Co., Ltd., Suzhou, China).

### 2.8. Statistical Analysis

All data are expressed as means ± standard deviations and were statistically analyzed by one-way analysis of variance (ANOVA) and least significant difference post hoc test using SPSS version 16.0 software (SPSS Inc., Chicago, IL. USA). Values with *P* < 0.05 were considered to differ statistically and significantly.

## 3. Results

### 3.1. Growth of HCT-8 and HCT116 Cells Was Inhibited by QFG

**Figure 1:**
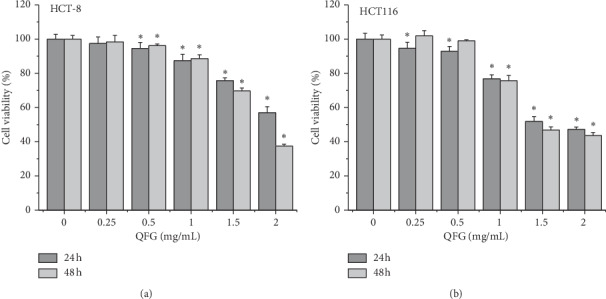
The effect of QFG treatment on HCT-8 and HCT116 cell growth. HCT-8 and HCT116 cells were incubated with QFG (0, 0.25, 0.5, 1, 1.5, or 2 mg/mL) for 24 or 48 h; then their growth was measured via the MTT assay. Data were normalized to the growth of untreated cells and are represented as the means ± standard deviations of three independent experiments. ^*∗*^*P* < 0.01 vs. untreated cells.

The growth-inhibitory effect of QFG on CRC cell lines of HCT-8 and HCT116 was measured via MTT assays.  Figure 1 shows that the viability of HCT-8 and HCT116 cells dose-dependently decreased following incubation with QFG for 24 and 48 h. The half-maximal inhibitory concentration (IC_50_) values of QFG at 24 and 48 h were, respectively, 2.583 and 1.849 mg/mL in HCT-8 cells, and, respectively, 1.797 and 1.608 mg/mL in HCT116 cells. These results suggest that QFG remarkably suppressed the growth of HCT-8 and HCT116 cells.

### 3.2. Migration and Invasion Abilities of HCT-8 and HCT116 Cells Were Suppressed by QFG

**Figure 2:**
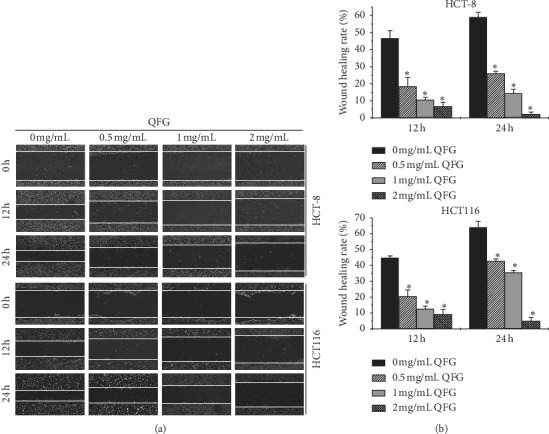
The effect of QFG treatment on HCT-8 and HCT116 cell migration using a wound healing assay. HCT-8 and HCT116 cells were incubated with QFG (0, 0.5, 1, or 2 mg/mL) for 24 h. (a) Images of wound healing captured at 0, 12 h, and 24 h using a phase contrast microscope at a magnification of 100×. (b) Data are represented as the means ± standard deviations of three independent experiments. ^*∗*^*P* < 0.01 vs. untreated cells.

**Figure 3:**
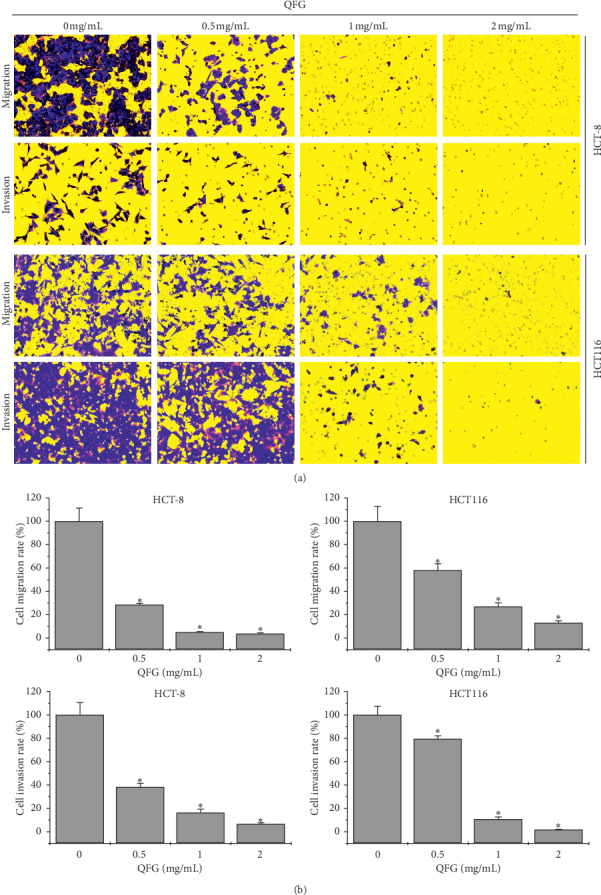
The effect of QFG treatment on HCT-8 and HCT116 cell migration and invasion using the Transwell assay. HCT-8 and HCT116 cells were incubated with QFG (0, 0.5, 1, and 2 mg/mL) for 24 h. (a) Average numbers of migrated and invasive cells in three random fields in the images of Transwell chambers at a magnification of 200×. (b) Data were normalized to untreated cells and are represented as the means ± standard deviations of three independent experiments. ^*∗*^*P* < 0.01 vs. untreated cells.

To better evaluate the function of QFG in the metastasis of CRC, wound healing and Transwell assays were carried out on HCT-8 and HCT116 cells. The results of wound healing suggested that the doses of QFG at 0 to 2 mg/mL could significantly decrease the rate of wound healing in HCT-8 and HCT116 cells from 46.53 ± 4.66 to 6.69 ± 2.51% and 44.74 ± 1.29 to 9.12 ± 3.01% in 12 h, and by 58.93 ± 2.97 to 2.10 ± 1.30% and 64.02 ± 3.79 to 4.86 ± 2.21% in 24 h, respectively (Figure 2). Similarly, the results of the Transwell assay was consistent with the wound healing assay, showing the migratory rate decreases from 28.51 ± 1.20 to 3.99 ± 0.19% and 57.98 ± 5.62 to 12.85 ± 1.82% in HCT-8 and HCT116 cells, respectively (Figure 3). Accordantly, incubated with 0.5, 1, 2 mg/mL QFG for 24 h dose-dependently decreased the invasion rate of HCT-8 and HCT116 cells from 38.19 ± 3.29 to 6.46 ± 0.45% and 79.42 ± 2.69 to 1.40 ± 0.06%, respectively, relative to the untreated cells (Figure 3). These results suggest that QFG played a suppressive role in the migration and invasion of HCT-8 and HCT116 cells.

### 3.3. QFG Inhibited TGF-*β*1/Smad Signaling Pathways in HCT-8 and HCT116 Cells

**Figure 4:**
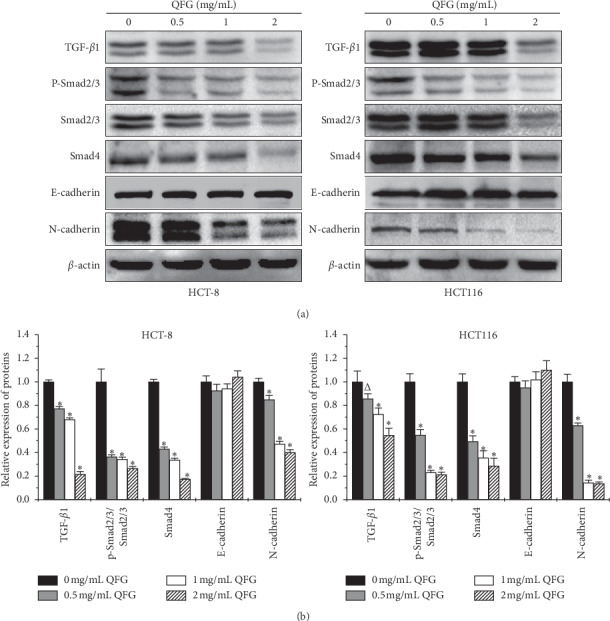
The effect of QFG treatment on the expression of proteins in the TGF-*β*1/Smad signaling pathway in HCT-8 and HCT116 cells. HCT-8 and HCT116 cells were incubated with QFG (0, 0.5, 1, or 2 mg/mL) for 24 h. (a) Protein expression of TGF-*β*1, p-Smad2/3, Smad2/3, Smad4, N-cadherin, and E-cadherin, as determined by western blotting. The internal control used was *β*-actin. Images are representative of three independent experiments. (b) Relative densitometric analysis of the above protein is displayed. ^Δ^*P* < 0.05, and ^*∗*^*P* < 0.01 vs. untreated cells.

The TGF-*β*1 signaling pathway is closely involved in the process of EMT [18, 19]. In order to explore whether QFG suppressed the TGF-*β*1/Smad signaling pathway, the TGF-*β*1 and several pivotal downstream proteins, e.g., p-Smad2/3, Smad2/3, and Smad4, were identified in HCT-8 and HCT116 cells via western blot analysis.  Figure 4 shows that QFG dose-dependently downregulated the expression of TGF-*β*1, p-Smad2/3, and Smad4 in both HCT-8 and HCT116 cells as compared to untreated cells. N-cadherin and E-cadherin are both downstream targets of the TGF-*β*1/Smad signaling pathway. A switch from E-cadherin to N-cadherin indicates the progression of EMT and further promotes the metastasis of cancers. Our results also showed that QFG reduced the expression of N-cadherin but did not significantly affect that of E-cadherin (Figure 4). This indicated QFG suppressed the cells EMT by decreasing the ratio of N-cadherin to E-cadherin. These findings indicate that the antimetastatic effect of QFG on CRC was at least partly associated with the inhibition of EMT mediated by the TGF-*β*1/Smad signaling pathway.

### 3.4. QFG Decreased lncRNA ANRIL Expression and Increased Let-7a Expression in HCT-8 and HCT116 Cells

**Figure 5:**
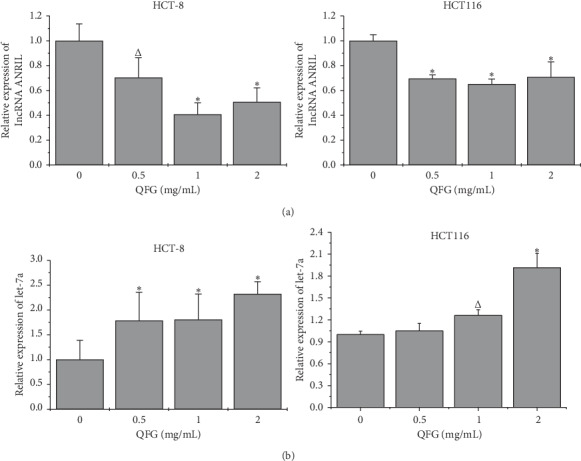
The effect of QFG treatment on the expression of lncRNA ANRIL and let-7a in HCT-8 and HCT116 cells. HCT-8 and HCT116 cells were incubated with QFG (0, 0.5, 1, or 2 mg/mL) for 24 h. (a) The expression of lncRNA ANRIL was determined using real-time PCR with GAPDH as the internal control. (b) The expression of let-7a was determined using real-time PCR with U6 as an internal control. ^Δ^*P* < 0.05, and ^*∗*^*P* < 0.01 vs. untreated cells.

LncRNA ANRIL acts as an oncogene that enhances cancer cell proliferation, disturbs the sensitivity of cancer cells to chemotherapeutic drugs, and promotes cancer cell migration and invasion [5, 20]. In contrast, let-7a inhibits cancer cell growth and metastasis through its target genes [21, 22]. Knockdown of lncRNA ANRIL increased the let-7a expression and further blocked the TGF-*β*1/Smad signaling pathway, which led to decreasing the migration of prostate cancer cells [7]. Therefore, to further explore whether QFG decreased CRC metastasis by regulating the lncRNA ANRIL/let-7a/TGF-*β*1/Smad axis, we performed RT-qPCR analysis to detect the expression of lncRNA ANRIL and let-7a.  Figure 5 shows that the expression of lncRNA ANRIL decreased, whereas that of let-7a increased in HCT-8 and HCT116 cells after treatment with QFG, compared with untreated cells. These findings were consistent with the inhibition of the TGF-*β*1/Smad pathway mediated by QFG (Figure 4). Taken together, these results suggest that the lncRNA ANRIL/let-7a/TGF-*β*1/Smad axis might be one of the underlying mechanisms through which QFG reduced HCT-8 and HCT116 cell migration and invasion.

## 4. Discussion

QFG is a TCM formulation, which consists of *Oldenlandia diffusa* (Willd.) Roxb., *Astragalus membranaceus* (Fisch.) Bge. var. *mongholicus* (Bge.) Hsiao, *Hordeum vulgare* L., *and Scutellariae Barbatae* D. Don. Our previous studies showed that *Oldenlandia diffusa* (Willd.) Roxb. inhibited 5-FU-resistant CRC cell metastasis by regulating the TGF-*β*1 signaling pathway [23]. A combination of *Astragalus membranaceus* (Fisch.) Bge. var. *mongholicus* (Bge.) Hsiao polysaccharide and 10-hydroxycamptothecin could inhibit nonsmall cell lung carcinoma metastasis via the MAP4K3/mTOR signaling pathway [24]. Total flavonoids of *Scutellariae Barbata*e D. Don have been reported to inhibit invasion of hepatocellular carcinoma via matrix metalloproteinases (MMP)/metalloproteinases (TIMP) and inhibit human breast carcinoma bone metastasis by inhibiting the parathyroid hormone-related protein (PTHrP) pathway [25, 26]. Thus, most of these compounds show powerful antimetastatic effects, which could explain why QFG inhibited the HCT-8 and HCT116 cells migration and invasion in our study.

Indeed, multiple signaling pathways are associated with cancer metastasis including MAPK (ERK1/2, JNK, p38), PI3K/AKT, NF-*κ*B, TGF-*β*, chemokine pathways, Grb2 and other adaptor protein pathways, and many others [27–30]. The present study found that QFG significantly inhibited the expression of several key proteins in the canonical TGF-*β*1/Smad pathway, including TGF-*β*1, p-Smad2/3, and Smad4. This inhibition led to a decrease in the ratio of N-cadherin to E-cadherin, which is considered as evidence of EMT inhibition [31].

The downstream TGF-*β*1/Smad pathway is one of several pathways that are mediated by the let-7a or lncRNA ANRIL. Overexpression of let-7a has an antimetastatic effect on glioma cells by negatively regulating the TGF-*β*1/Smad3 signaling pathway [32]. Nonetheless, Silvia Ottaviani et al. provided further evidence that TGF-*β*1 may also be the upstream signaling pathway to block let-7a expression in the progression of pancreatic ductal adenocarcinoma [33]. Collectively, the relationship between the TGF-*β*1/Smad signaling pathway and let-7a is negative. However, accumulating evidence indicates that lncRNA ANRIL could increase the growth and migration and invasion abilities of cancer cells by positively regulating the TGF-*β*1/Smad signaling pathway in oral squamous cell carcinoma, thyroid cancer, and esophageal squamous cell carcinoma [34–36]. Many studies have reported the interactions among lncRNA ANRIL-let-7a-TGF-*β*1/Smad through competing endogenous RNA (ceRNA) networks that control the development of cancer. Silencing of lncRNA ANRIL significantly decreases prostate cancer cell proliferation and migration by increasing let-7a expression and further blocking the TGF-*β*1/Smad signaling pathway [7]. We found increased let-7a and decreased lncRNA ANRIL expression after incubating HCT-8 and HCT116 cells with QFG treatment, which is consistent with the notion that QFG induces a blockade of TGF-*β*1/Smad signaling.

**Figure 6:**
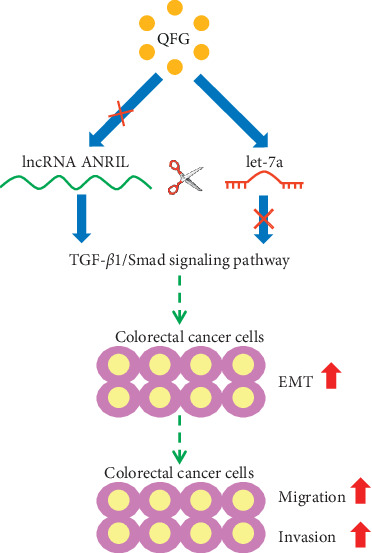
A proposed model for illustrating the possible role of QFG on the migration and invasion of colorectal cancer cells.

In summary, the present study uncovered evidence showing that QFG inhibits the growth and migration and invasion abilities of human CRC cells by regulating the ceRNA network of lncRNA ANRIL-let-7a-TGF-*β*1/Smad (Figure 6). Our findings provide a solid scientific basis for QFG as an adjunctive medicine in the treatment of CRC clinically.

## Data Availability

The datasets used and/or analyzed during the current study are available from the corresponding author upon reasonable request.

## Abbreviations

CRC: Colorectal cancer
QFG: Qingjie Fuzheng granule
TCM: Traditional Chinese medicine
EMT: Epithelial-mesenchymal transition
TGF-*β*1: Transforming growth factor-*β*1
TGF-*β*R I: TGF-*β*1 receptor type I
IC_50_: Half-maximal inhibitory concentration
MMP: Matrix metalloproteinases
TIMP: Metalloproteinases
PTHrP: Parathyroid hormone-related protein
ceRNA: Competing endogenous RNA.
lncRNA ANRIL: LncRNA antisense noncoding RNA in the INK4 locus.
5‐FU‐: 5‐fluorouracil‐.
